# *Trichoderma* spp.-mediated mitigation of heat, drought, and their combination on the Arabidopsis thaliana holobiont: a metabolomics and metabarcoding approach

**DOI:** 10.3389/fpls.2023.1190304

**Published:** 2023-08-21

**Authors:** Biancamaria Senizza, Fabrizio Araniti, Simon Lewin, Sonja Wende, Steffen Kolb, Luigi Lucini

**Affiliations:** ^1^ Department for Sustainable Food Process, CRAST Research Centre, Università Cattolica del Sacro Cuore, Piacenza, Italy; ^2^ Dipartimento di Scienze Agrarie e Ambientali, Produzione, Territorio, Agroenergia (Di.S.A.A.) Università degli Studi di Milano, Milano, Italy; ^3^ Research Area Landscape Functioning, Leibniz Center for Agricultural Landscape Research – ZALF, Munchenberg, Germany; ^4^ Thaer Institute, Faculty of Life Sciences, Humboldt University of Berlin, Berlin, Germany

**Keywords:** climate change, combined stress, abiotic stress, biostimulants, rhizosphere microbiome, meta-barcoding, multi-omics

## Abstract

**Introduction:**

The use of substances to increase productivity and resource use efficiency is now essential to face the challenge of feeding the rising global population with the less environmental impact on the ecosystems. *Trichoderma*-based products have been used as biopesticides, to inhibit pathogenic microorganisms, and as biostimulants for crop growth, nutrient uptake promotion, and resistance to abiotic stresses.

**Methods:**

In this work, plant metabolomics combined with roots and rhizosphere bacterial metabarcoding were exploited to inspect the performance of *Trichoderma* spp. biostimulants on *Arabidopsis thaliana* under drought, heat and their combination and its impact on plant holobiont.

**Results and discussion:**

An overall modulation of N-containing compounds, phenylpropanoids, terpenes and hormones could be pointed out by metabolomics. Moreover, metabarcoding outlined an impact on alpha and beta-diversity with an abundance of *Proteobacteria*, *Pseudomonadales*, *Burkholderiales*, *Enterobacteriales* and *Azospirillales*. A holobiont approach was applied as an integrated analytical strategy to resolve the coordinated and complex dynamic interactions between the plant and its rhizosphere bacteria using *Arabidopsis thaliana* as a model host species.

## Introduction

Heat and drought are the two major stresses impacting the growth and productivity of crops. These stresses normally occur together because of the rapid water loss from the soil due to increased temperatures, and their effects are more severe than those of individual stress. Heat and drought combination negatively affect plant physiological processes such as photosynthesis and respiration, life cycle and reproductive stages leading to severe consequences for food production and quality. In this scenario, the use of substances able to improve crop productivity and increase plant stress resistance with minimal environmental impacts has become fundamental. The plants’ morpho-physiological and molecular mechanisms involved in the response to co-occurring abiotic stresses are often shared, paving the way to synergistic or interactive molecular mechanisms (Mittler, 2006; Mahmood et al., 2021). Although being now widely recognized that abiotic stress combination is not merely the sum of individual stresses (Rötter et al., 2018; Webber et al., 2022), little is known about the role of mitigation measures under multiple stress conditions.

Among sustainable approaches to cope with stresses, microbial-based biostimulants have been reported as functional thanks to their beneficial effects on crops (Del Buono, 2021; Rai et al., 2021; Franzoni et al., 2022) under abiotic stresses like salinity, heat, and drought, as well as able to elicit resistance to plant pathogens (Fiorentino et al., 2018). *Trichoderma* spp. is a genus of filamentous fungi possessing the ability to colonize diverse substrates and interact with plants; this interactions lead to significant shifts in plant metabolism, hormone production, soluble sugars, phenolic compounds, and amino acid content (Iula et al., 2021). Under different environmental conditions, *Trichoderma* spp. can establish symbiotic relationships with a wide range of host plants, secrete hydrolytic enzymes and secondary metabolites and improve biotic and abiotic stress tolerance and plant defenses and protection (Nicolás et al., 2014). When applied to leaves, soil or seeds, the beneficial effects on plant defense and growth, root development, activation of seed germination, chlorophyll content and photosynthetic efficiency (Abdullah et al., 2021) are reported. Thus, this association can help plants to sustain drought stress by increasing the expression of antioxidative enzymes, regulating the absorption surface (Bashyal et al., 2021) and adapting the synthesis of phytohormones to promote plant performances (Illescas et al., 2021). During high temperature, the fungi ameliorate the detrimental effect of the stress modulating the antioxidative network, decreasing the hydrogen peroxide production, reducing the ROS content, and increasing the activity of the phenylalanine ammonia lyase (Tripathi et al., 2021). In addition, *Trichoderma* spp. secondary metabolites induce root branching and increase shoot biomass thanks to the auxin-like or auxin inducer compounds which facilitate root exudates and signaling molecules exchange between the fungus and the plant. Through correlation-based analysis of untargeted metabolomics and metabarcoding, an integrated knowledge about the metabolic networks that regulate plant stress responses and how plant secondary metabolites can change plant microbiomes could be achieved. Also, the capabilities of microbiomes to influence important plant traits such as growth, abiotic stress tolerance, resistance to infectious diseases, and the synthesis of phytohormones might be established.

On these bases, our work aimed at investigating the *Trichoderma*-mediated impact of heat, drought, and their combination on the plant holobiont, considering physiological responses, molecular implications at metabolome level, as well as the root and rhizosphere bacterial microbiota. Recently, a holo-omics approach has been suggested to assess, simultaneously in one experimental design, both the plant host and its microbiota response to environmental changes to better understand modified interactions and the relevance of enriched microbial taxa for its host plant (Xu et al., 2021).

## Materials and methods

### Plant growth conditions and treatments

The experiment was conducted on *Arabidopsis thaliana* plants (cv Columbia 0) according to the protocol proposed by Rizhsky et al. (2004) with some modifications. As substrate, professional potting soil (orticole alveolo TecnoGrow, Tercomposti, Italy, Calvisano, BS), sterilized before the experiment started at 120° per 20 min, was used. Before sowing, seed germination was synchronized by soaking the seeds in sterile water for 76 hours at 4 ° C in dark conditions. Ten seeds were then sown per pot; after germination, they were thinned, leaving one seedling per pot. Twenty-four pots (5 cm X 5 cm X 5 cm) for each treatment and replicate. Seedlings were grown in a growth chamber under controlled conditions: 12 light / 12 dark photoperiod (long day), 21±1°C, 100 µmol m^-2^ s^-1^, and relative humidity of 60 % - 70%. Seedlings were fertilized every other day through sub-irrigation using a half-strength Hoagland solution. Then they were grown for 22 days [Arabidopsis growth stage 3.50, Rosette is ~ 50% final size (Boyes et al., 2001)], when the commercial formulation Condor Shield (*Trichoderma koningii* TK7, 1 × 109 CFU/g, Atens - Agrotecnologias Naturales SL, Tarragona, Spain), at the label-recommended application rate (200 mg/mL), was applied on leaf tissue. The treatment was applied once, six days before the stress induction, when the *Arabidopsis thaliana* growth stage was 3.90, and the rosette growth almost complete (Boyes et al., 2001)]. The following treatments have been applied: C (Control watered plants), CT (Control + *Trichoderma*), CT+D (*Trichoderma* + drought), CT+H (*Trichoderma* + Heat), CT+H+D (*Trichoderma* + Heat + Drought). Drought treatment was achieved by blocking plant irrigation until they reached a relative water content (RWC) of 65 % to 70 % (typically 5–6 d). In contrast, heat stress was applied by raising the temperature gradually (~ 4 °C per hour) to avoid a too aggressive heat shock in the growth chamber to 35 °C and then keeping the plants at this temperature for 12 h. At the end of the experiments, 1 g of the rhizosphere and root samples were collected for the microbiome analysis. Roots were carefully washed in sterilized distilled water and immediately snapped frozen in liquid nitrogen. Both the collected soil and extracted powdered roots were all stored at -80 °C.

### Morpho-physiological assays

During the experiments, morpho-physiological parameters were monitored in pre and post-harvest. In particular, the chlorophyll content (using a chlorophyll meter SPAD-502 Minolta, Milan, Italy) and the photosystem II (PSII) efficiency of the dark-adapted leaves (using the fluorescence spectrometer MultispeQ V 2.0 PhotosynQ, East Lansing, MI, USA) were monitored in pre-harvest. Moreover, leaf temperature was monitored using a thermo-camera (FLIR T640bx) and analyzed using the Flir Tool software. After collection, samples were wrapped in aluminum paper to evaluate fresh weight (FW) and then transferred in an oven (60 °C x 72h) to calculate dry weight (DW) and DW/FW ratio.

### Untargeted metabolomics analysis

*Arabidopsis* roots were extracted in methanol:water solution (80:20) with 0.1% (v/v) formic acid using a homogenizer-assisted extraction, centrifuged and filtered through 0.22 µm cellulose filters. As previously reported by Senizza et al., 2021, the phytochemical profile of roots was investigated according to an untargeted metabolomics approach. To this aim, ultra-high-performance liquid chromatography (UHPLC) coupled with quadrupole-time-of-flight (QTOF) mass spectrometry (1290 UHPLC and 6550 iFunnel QTOF MS, both from Agilent technologies, Santa Clara, CA, USA) were used. The mobile phase consisted of a mixture of water and acetonitrile (both LC-MS grade, VWR, Milan, Italy) acidified with 0.1% (v/v) formic acid, with a gradient from 6 to 94% of acetonitrile in 35 min. An injection volume of 6 μl was chosen and a pentafluorophenylpropyl column (2.0 × 100 mm, 3 µm particle size - Agilent technologies) was used for chromatographic separation. The mass spectrometer acquired ions in the range 100-1200 m/z in positive scan mode (ESI+) at a rate of 0.8 spectra/s (40,000 FWHM, absolute peak height threshold 5000 counts).

The software Profinder B.07 (Agilent Technologies) was used for raw data processing. The monoisotopic mass (5-ppm tolerance for mass accuracy), isotope spacing, and ratio were considered according to the “find-by-formula” algorithm. Before the compound’s annotation, mass, retention time alignment, and compound filtering were performed. The database PlantCyc was used as a reference for annotations (Hawkins et al., 2021), and only those compounds identified in 100% of the replications within at least one condition were retained in the dataset and considered further. According to COSMOS Metabolomics Standards Initiative, the annotation process corresponded to a Level 2 of identification (i.e., putatively annotated compounds) (Salek et al., 2015).

### Analysis of the rhizosphere bacterial microbiota structure by metabarcoding

The DNeasy PowerLyzer PowerSoil Kit (Qiagen, Hilden, Germany) was used to extract DNA from roots and soil samples. The amplification of bacterial DNA was carried out by LGC genomics GmbH (Berlin, Germany) using the primers 799f and 1115r and the amplicons were sequenced on an Illumina MiSeq instrument with 300bp paired-end reads.

Demultiplexing was conducted with Illumina bcl2fastq 2.17.1.14 software following the clipping of barcode and sequencing adapters. Primers were removed using Cutadapt v3.0 (Martin, 2011). Sequences were processed in R 4.1 with dada2 version 1.22.0 (Callahan et al., 2016). Due to adapter ligation-based library prep, the raw sequences were in mixed orientation. To get the correct final orientation for learning error rates, reads were split into two groups (forwardRead.forwardPrimer - reverseRead.reversePrimer, and reverseRead.forwardPrimer - forwardRead.reversePrimer), denoised separately and merged after chimera removal. Forward and reverse reads were truncated at positions 265 and 210, resulting in 4073 unique Amplicon sequencing variants (ASV). Taxonomic classification was performed using the q2-feature-classifier plugin from Qiime2 version 2021.8.0 with a naïve Bayes classifier trained on the Silva 138.1 NR99 database.

### Statistical analysis

All the experiments were carried out in a completely randomized design with 5 replications. The univariate analysis of morphological and physiological parameters was carried out using XLSTAT 2014.5.03. Data were analyzed through one-way ANOVA using Duncan’s test as *post hoc* (*p*≤ 0.05). Concerning metabolomics, the post-acquisition data analysis was carried out using the software Mass Profiler Professional 12.6 (Agilent Technologies); the compounds were log2 transformed, normalised at the 75th percentile, and baselined against the median. Afterwards, both unsupervised and supervised multivariate statistics were applied for interpretations. According to Euclidean distance and Ward's linkage, the unsupervised hierarchical cluster analysis was used to naively point out patterns across the different treatments. In addition, the supervised orthogonal projection to latent structures discriminant analysis (OPLS-DA) was carried out, and the model parameter (goodness-of-fit R^2^Y and goodness-of-prediction Q^2^Y) were recorded. Also, the OPLS-DA model was cross-validated (CV-ANOVA), inspected for outliers (Hotelling’s T2) and the overfitting was excluded through a permutation test (n = 100). Then, the Variable Importance in Projection (VIP) analysis was used to select the metabolites having the highest discriminant potential (VIP score > 1.2). Finally, the differential compounds obtained from the ANOVA and fold-change analysis (FC) (*p* < 0.05, Bonferroni multiple testing correction and Fold-Change FC ≥ 2) were exported into the Omic Viewer Pathway Tool of PlantCyc (Plant Metabolic Network) (Stanford, CA, USA) software for interpretation (Karp et al., 2009; Caspi et al., 2013).

Regarding metabarcoding data, downstream analysis was performed using RStudio with R version 4.1.1. Phyloseq v1.38.0 was used to handle ASV sequences and tables. Samples were split into compartments (soil, root) and analysed separately. The following filtering steps were applied during the analysis: first ASVs were filtered for mitochondria, and unassigned sequences were removed. Samples were rarefied to minimum sample size (= 17413 total sequences). When filtering was applied, only ASV that (a) occurred in at least 3 samples and (b) occurred >10 times in total were retained for downstream analysis. Alpha diversity indices (number of observed ASVs, Inverse Simpson index) were calculated using rarefied samples from root and soil datasets and plotted by treatment. Bray-Curtis dissimilarity indices were calculated on Hellinger transformed abundances and used to perform principal coordinate analysis (PCoA) and permutational analysis of variance (PERMANOVA) with 999 permutations to investigate treatments' effect on the bacterial community structure.

Linear discriminant analysis of effect size (LEfSe) was applied to the root and soil datasets to identify keystone taxa that drive the differences between treatments (Segata et al., 2011). LEfSe was run with a Wilcoxon and Kruskal-Wallis cut-off value of 0.01, ASV counts were log-transformed with pseudocounts (log10p) and normalized per sample to the sum of value to 1e+06 (CPM). An LDA cut-off value of 2 resulted in 154 and 191 marker genera for root and soil, respectively. In addition, analysis of compositions of microbiota with bias correction (ANCOMBC) was used to identify differentially abundant features (Lin & Peddada, 2020). ASV counts were log10p transformed, normalized to 1e+06 (CPM), and the Holm-Bonferroni method was applied to adjust *p*-values. Only features with adjusted *p*-values < 0.01 were considered significant, resulting in 106 and 139 markers for root and soil datasets, respectively.

### Combined discriminant analysis of metabarcoding and metabolomics datasets

Data Integration Analysis for Biomarker discovery using Latent variable approaches for Omics studies (DIABLO) from the package “mixOmics” was used for the integration of metabolomics and root metabarcoding datasets (Rohart et al., 2017). This supervised approach allowed the integrated analysis of multiple datasets and was used to identify discriminant features in both datasets that drive differences between treatment groups. Values of the design matrix were set to 0.1 to prioritise the discriminant ability of the model. Center log ratio (clr) transformation was applied to both datasets, and root metabarcoding data was aggregated at the genus level beforehand. An optimal number of 4 components for “centroid.dist” distance was determined using the function perf with a 4-fold cross-validation and 10 repeats. The number of features selected for sparse PLS-DA was tuned with the function tune.block.splsda using 4-fold cross-validation with 10 repeats. The features selected for each component were 34, 40, 18 and 10 for metabarcoding and 6, 14, 70 and 40 for metabolomics. The correlation among components of each dataset was checked with plotArrow.

## Results

### Morpho-physiological parameters of *A. thaliana*

**Figure 1:**
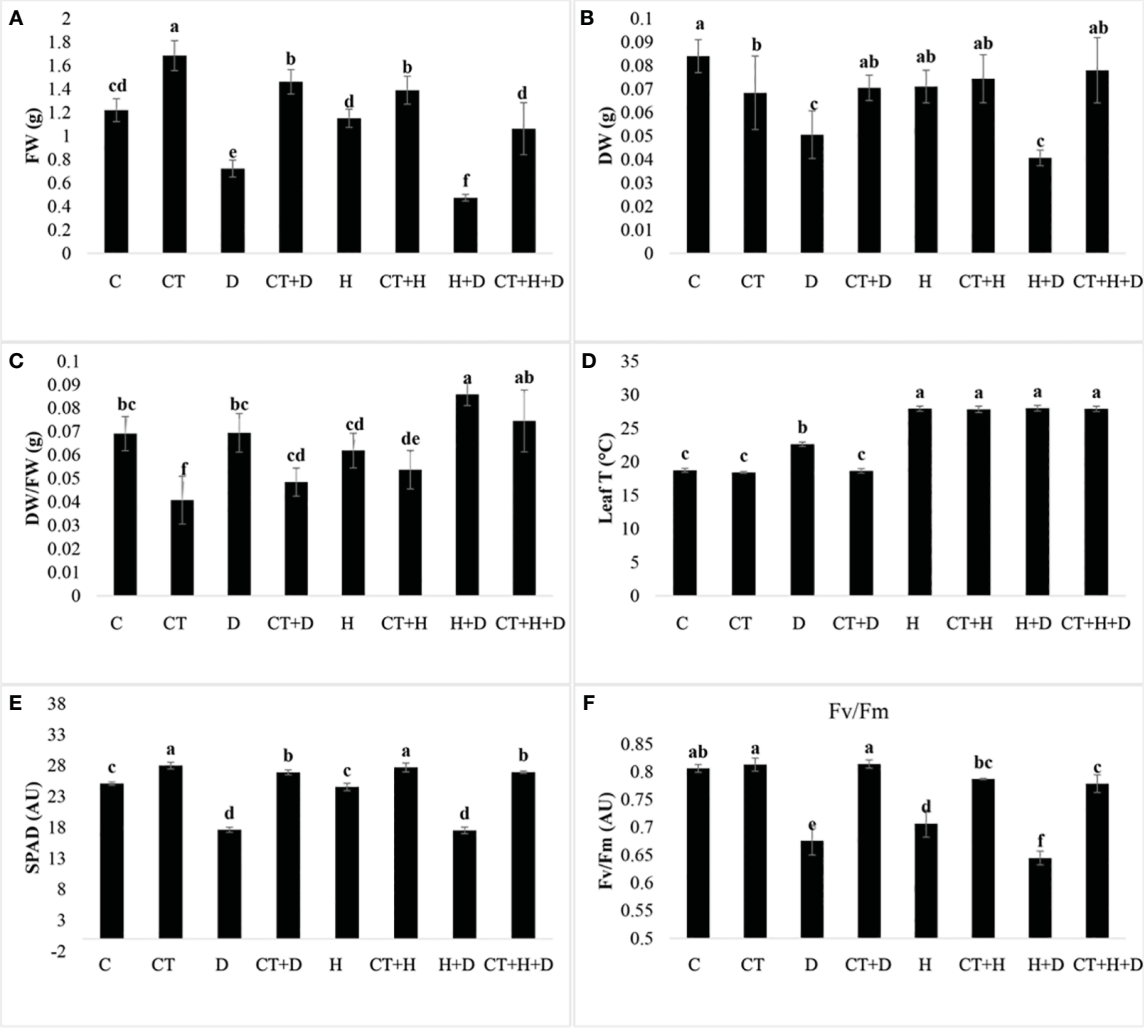
Effects of single and combined stress on adult plants of Arabidopsis thaliana in control conditions or treated with Trichoderma before stress application. Fresh weight (FW), dry weight (DW), their ratio (FW/DW), leaf temperature (leaf T), SPAD index (SPAD) and photosystem II efficiency (Fv/Fm) are provided from panel **(A–F)** for the following treatments: C (untreated control); CT (control treated with Trichoderma); D (untreated control + Drought stress), CT+D (CT + drought stress); H (untreated control + Heat stress), C+H (untreated control + heat stress), CT+H (CT + heat stress); H+D (untreated control + stress combination), CT +H+D (stress combination). Data were analyzed through two-way ANOVA using the LSD’s test as *post-hoc*. Different letters along the bars indicate statistical differences with *p*≤0.05 (N=4).

The biostimulant was applied six days before the stress induction. The trial was assessed to evaluate *Trichoderma* performance in mitigating the stresses. Treatments without *Trichoderma* spp. revealed that all the plant physiological parameters were significantly affected by the stresses (D, H, D+H). The ratio of plant dry versus wet weight was only affected by stress treatments (**Supplementary Table S1**). These data highlighted that in control plants, to which *Trichoderma* spp. was not applied, drought stress and its combination with heat (D+H) significantly reduced plant biomass. On the contrary, the application of *Trichoderma* spp. significantly stimulated the fresh weight in both control conditions and stress application (**Figure 1A**). Concerning dry weight, in control treatments with drought and its combination with heat reduced the monitored parameter, whereas the heat as stressor alone was not affective. *Trichoderma* spp. application slightly reduced plant dry weight, but allowed to maintain dry weight when the stressor drought and drought combined with heat were applied (**Figure 1B**). These results highlighted that *Trichoderma* spp. treatment was significantly reduced compared to control only when the stress was not applied (**Figure 1C**). In addition, considering only plants where *Trichoderma* spp. was applied (CT, CT+D, CT+H and CT+H+D), we observed a significant increase of this parameter (**Figure 1C**). The monitoring of leaf temperature highlighted that during drought stress, *Trichoderma* spp. application allowed to maintain a lower temperature compared to the control. On the contrary, no differences were observed during heat stress and the combined stresses drought and heat (**Figure 1D**). The SPAD analysis also revealed a higher content of pigments in all the treatments to which *Trichoderma* spp. was applied. Whereas in control plants a general reduction induced by stresses was detected, i.e. drought was more effective in reducing SPAD units than heat (**Figure 1E**).

Finally, *Trichoderma* spp. treatment significantly protected the Fv/Fm parameter which on the contrary was significantly reduced in all the stress treatments without *Trichoderma* spp. (**Figure 1F**).

### *Arabidopsis thaliana* roots profiling through UHPLC/QTOF-MS untargeted metabolomics

The untargeted metabolomics approach allowed us to putatively annotate 3000 compounds further used to investigate the role of *Trichoderma* spp. on *Arabidopsis thaliana* defense system and elucidate the biosynthetic processes involved. The whole list of annotated metabolites, composite mass spectra, and abundance are listed in the supplementary material (**Table S1**). Firstly, the unsupervised hierarchical cluster analysis (HCA) was carried out. As shown by the heat map, two clusters were generated, one consisted of the drought stressed samples, and the other included two main sub-clusters. One cluster comprised the thermally stressed samples (CT+H) and the heat-drought combination (CT+H+D), while the other one included the not-treated and the *Trichoderma* spp. treated roots.

**Figure 2:**
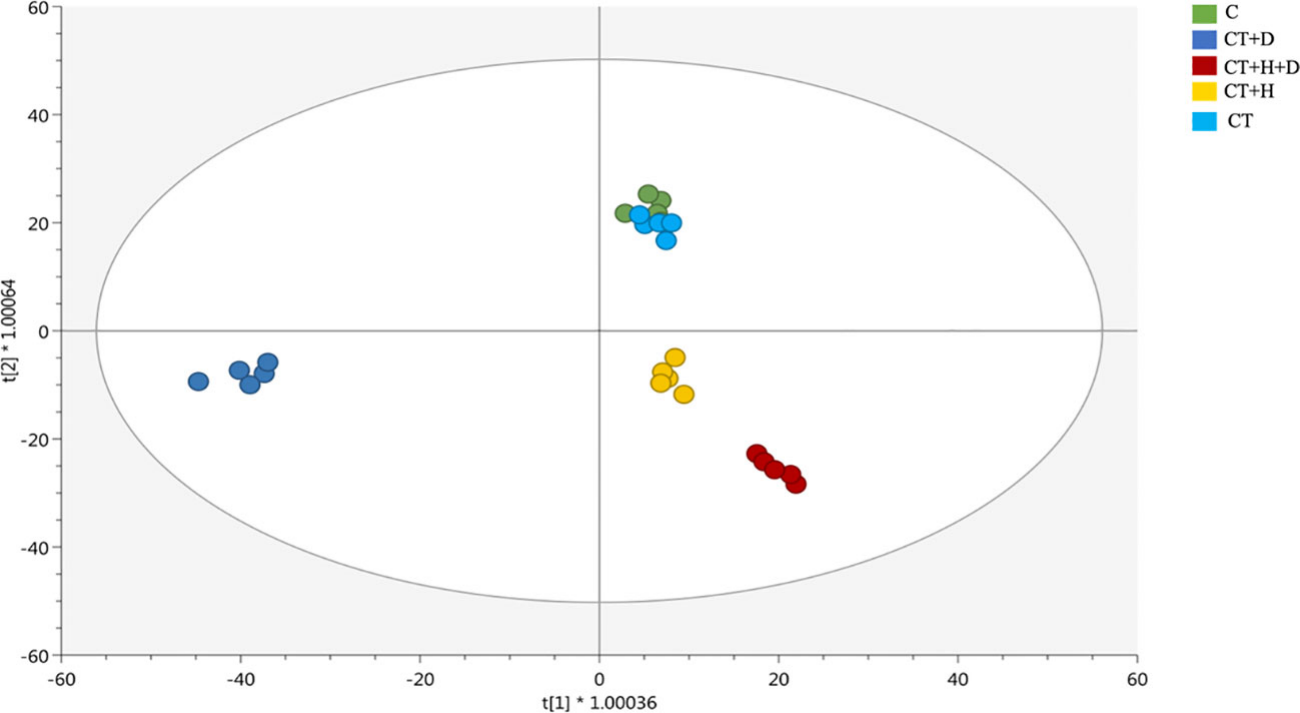
Score plot of orthogonal projection to latent structures discriminant analysis (OPLS-DA) supervised modeling carried out on untargeted metabolomics profiles of Arabidopsis roots exposed to heat, drought, and combined stresses (R2Y = 0.98, Q2Y = 0.93).

Afterwards, these results were further confirmed by the supervised OPLS-DA model revealing a more similar metabolic profile between the combined drought and heat stresses and the heat stress alone (**Figure 2**). On the first latent vector, a more distinctive profile revealed for the drought, while on the second vector, the separation between the stressed and non-stressed roots was evident. Interestingly, fungus affected the *Arabidopisis thaliana* chemical signature as detected as a separation between heat and drought alone and their corresponding controls (**Figure 2A**) and between all the treated samples and the non-treated ones based on the OPLS-DA score plot (**Figure S2B**).

Then, the model parameters were determined, being goodness-of-fit (R2Y cum) = 0.98 and goodness-of-prediction (Q2 cum) = 0.93, the permutation test cross-validation (N = 100) excluded the over-fitting, CV- ANOVA for significance testing showed a *p*-value of 1,85 × 10^-21^.

**Figure 3:**
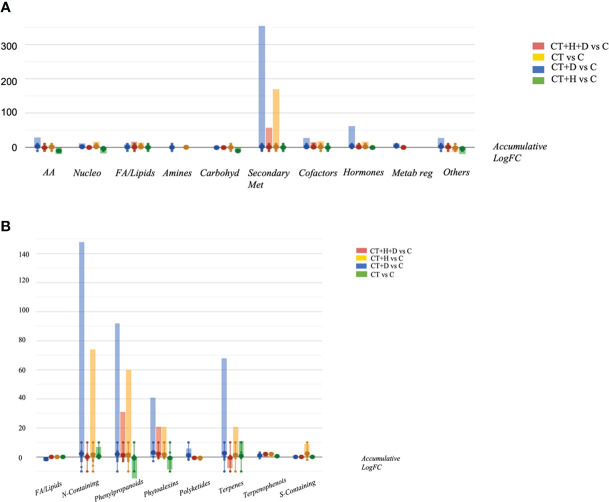
Metabolic processes **(A)** and specialised metabolite biosynthesis **(B)** modulated Arabidopsis thaliana roots treated with *Trichoderma* spp. and exposed to heat, drought and the combined stress (heat+ drought). The metabolomics dataset produced through UHPLC-ESI/QTOF-MS was subjected to ANOVA and FC analysis (*p* < 0.05, FC ≥ 2), and differential metabolites were loaded into the PlantCyc Pathway Tool (https://www.plantcyc.org/).

Looking at the OPLS-DA score plot obtained considering the stressed samples without *Trichoderma* spp. (**Figure S2**), it was clear that the fungus affected the chemical signature of the plants, as suggested by **Figure S2A** and B. Following, the VIP approach used to select the metabolites able to drive sample separation and most affected by the treatment identified 60 compounds with VIP score > 1.2 (**Table S2**). The compounds recording higher values belonged to terpenoids, phenylpropanoids and alkaloids. Finally, the Volcano analysis (*p≤0.05*; FC>2) was performed and the 492 distinguishable compounds were uploaded into PlantCyc pathway tools to provide insight into the effect of the stress treatment on roots' metabolic fingerprint (**Table S3**). The analysis caused a positive impact on the secondary metabolites, hormones, fatty acids, and amino acid synthesis in response to specific stress (**Figure 3A**). Overall, modulation of phenylpropanoid derivatives, N-containing metabolites (including alkaloids and polyamines), phytoalexins and terpenes was observed (**Figure 3B**). Regarding the nitrogen-containing compounds, a strong up accumulation was observed, especially in water deficit samples and, to a lesser extent, in CT+H and CT. Moreover, the synthesis of phenylpropanoids and phytoalexins was promoted by the CT+stresses, albeit the fungus alone revealed a general down accumulation of these two classes of metabolites. Also, a decrease in terpenes was highlighted in the stress combination samples, while an increase of these compounds was disclosed with single stress application and following *Trichoderma* spp. treatment.

**Figure 4:**
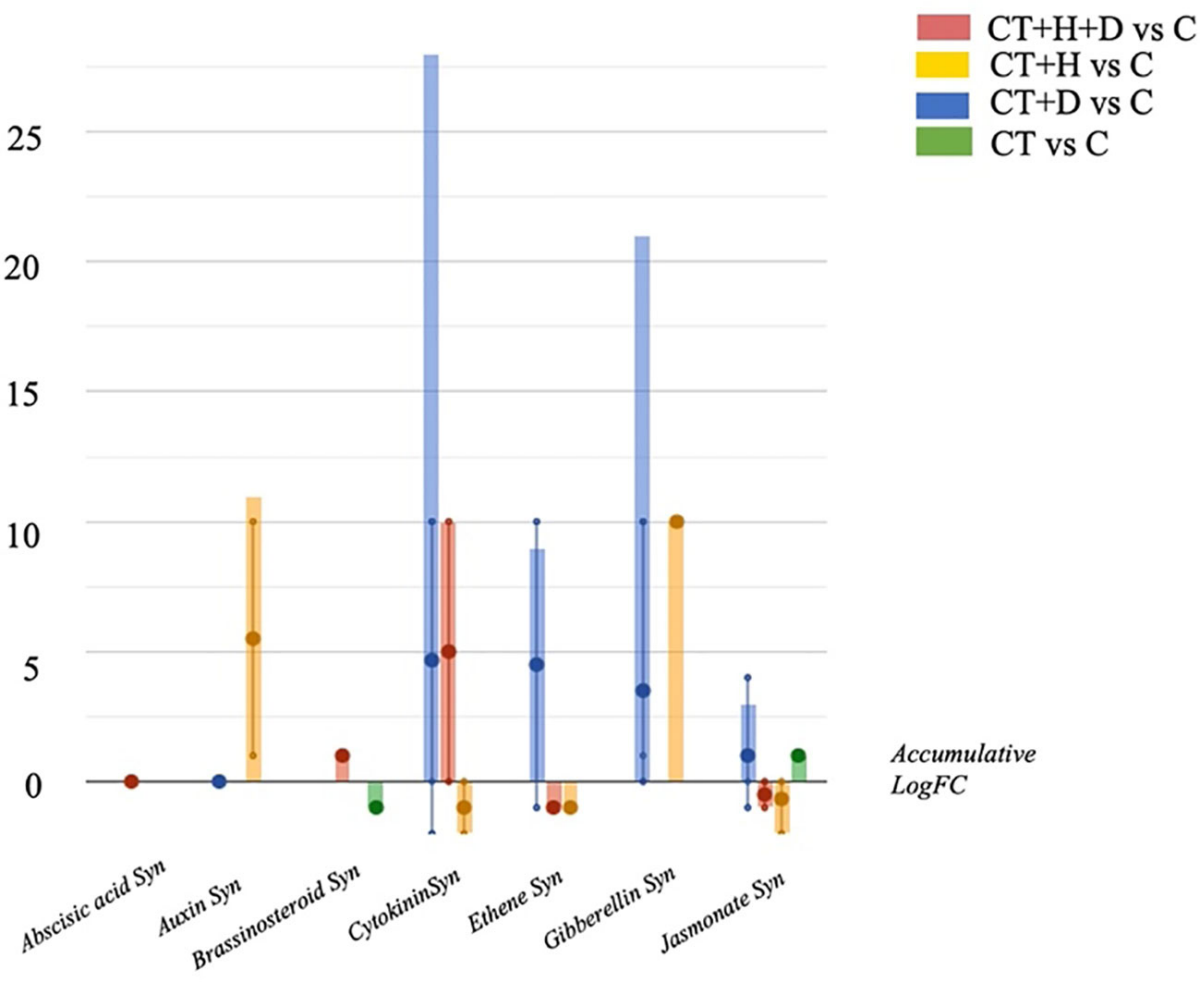
Hormones biosynthesis modulated in Arabidopsis thaliana roots treated with *Trichoderma* spp. and exposed to heat, drought, and the combined stress (heat+ drought). The metabolomics dataset produced through UHPLC-ESI/QTOF-MS was subjected to ANOVA and FC analysis (*p* < 0.05, FC ≥ 2), and differential metabolites were loaded into the PlantCyc Pathway Tool (https://www.plantcyc.org/)The x axis represents each set of metabolic subcategories, while the y axis corresponds to the accumulative log fold change (FC). The large dots represent the average (mean) of all FCs for the different metabolites in the class, while the small dots represent the individual log FC.

Therefore, the hormones profile was investigated, underlighting the accumulation of auxins, gibberellins, brassinosteroids and cytokinins depending on the stress considered (**Figure 4**). Above all, the accumulation of cytokinins and gibberellins and, to a lesser extent, jasmonates was observed in treated roots during drought conditions. During heat, the auxins and gibberellins synthesis were elicited while the cytokinins and jasmonates were down accumulated. The stress combination (CT+H+D) induced the increase of cytokinins and brassinosteroids but also affected the jasmonates, causing a reduction of their synthesis.

### Bacterial richness and biodiversity

**Figure 5:**
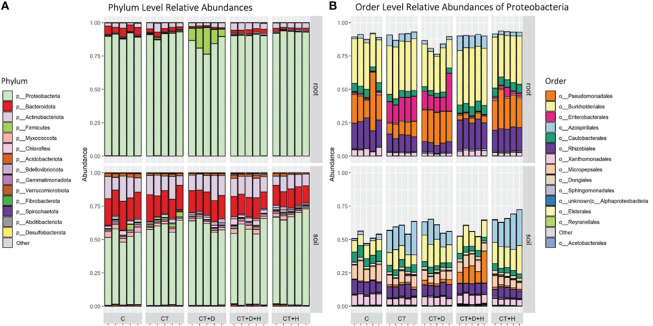
**(A)** Taxonomic Barplot of relative abundances for the top 14 most abundant Phyla in root and rhizosphere soil samples, grouped by treatments (C = Control watered plants, CT = Control + *Trichoderma*, CT+D = *Trichoderma* + Drought, CT+H = *Trichoderma* + Heat, CT+H+D = *Trichoderma* + Heat + Drought). **(B)** Taxonomic Barplot shows the 14 most abundant Orders within the dominating Phylum Proteobacteria.

After filtering for rare ASVs, 1171 and 733 bacterial ASVs were identified in soil and root samples, respectively, affiliated with 18 phyla, 35 classes, 80 orders, 128 families and 266 genera in the root, and 25 phyla, 48 classes, 111 orders, 182 families and 348 genera in soil. *Proteobacteria* were the most predominant in roots and soil, on average, 89.6% of roots and 59.4 % of soil (**Figure 5A**). Besides *Proteobacteria* in soil samples, one of the most abundant phyla were *Bacteroides* and *Actinobacteroides*, while in roots, the C seems to be characterized by a higher fraction of *Bacteroides*. In drought stressed roots, a relatively large fraction of Firmicutes (mean 11.8 %) were observed, almost all classified as *Clostridium sensu stricto 1.* Inspecting for the most abundant orders within the dominant Proteobacteria, differences between the two compartments were outlined (**Figure 5B**). In fact, in roots, we have larger fractions of *Burkholderiales, Pseudomonales*, *Enterobacterales*, and *Rhizobiales*, particularly when considering the CT and CT+D while in soil, a substantial fraction of *Azospirillales* (CT, CT+D and CT+H) and *Micopepsales* was underlined. Only one replicate related to C of the roots was dropped as an outlier from subsequent analyses, as it shows a highly different composition from other samples within this group and affected downstream analysis.

**Figure 6:**
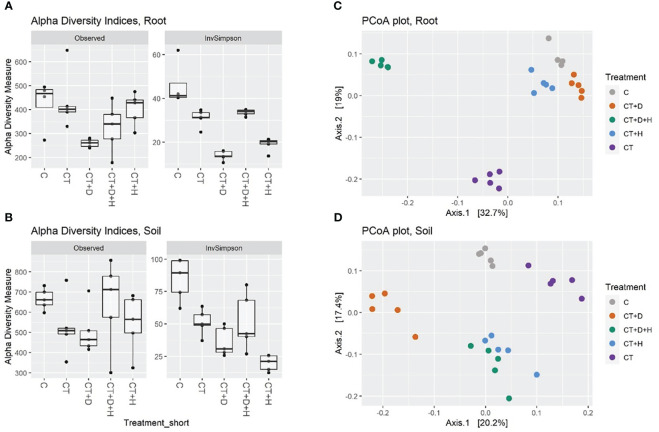
**(A, B)** show alpha diversities (as a number of Observed ASV and Inverse Simpson index) of root and soil samples. **(C, D)** show beta diversity, the filtered read counts were transformed by Hellinger transformation, PCoA was used for the ordination plot based on Bray-Curtis distances of samples.

In both root and soil samples, the general species richness (observed alpha diversity) is highest in control samples: the treatment application leads to a reduced number of species in a system (**Figure 6A, B**). As expected, the richness was higher in soil samples, with a great variation in the CT+H+D treatment, recording a richness comparable to the control. Moreover, reduced alpha diversity was fulfilled with *Trichoderma*'s application; both compartments were mostly affected by stress combination (CT+H+D). In addition, in drought samples, the highest loss in species number was underlined, but looking in detail at the Inverse Simpson index, which considers richness and evenness, it was noted that the heat treatment decreased similarly to the richness in the roots and more in the soil, indicating that, although species richness was not reduced a lot compared to control, the treatment leads to unevenness selection of dominating species. Afterwards, analysis of variance (ANOVA) followed by Tukey’s honestly significant difference (HSD) *post hoc* test revealed no effect of the treatments on the richness in soil and only a slight significance in the root (*p* ≤ 0.029). For the inverse Simpson index, a significant effect of the treatment was identified in root and soil (*p* ≤ 0.001)

The beta-diversity was determined using principal coordinate analysis (PCoA) with Bray-Curtis dissimilarities (**Figures 6C, D**). The first two axes accounted for 51.7 % and 37.6 % of the variance between treatments of root and soil samples, respectively. On the first two axes, a good grouping of all treated roots was observed, underlighting that CT and CT+H+D samples clustered farthest from the other treatments and CT+H, CT+D and C were closer. PERMANOVA analysis on root and soil datasets indicated a significant effect of all treatments (**Table S4**). In root samples, drought treatments explain 16% of the variance, heat 23%, *Trichoderma* treatment 20%, and the combination an additional 18 %. In soil samples CT+D explained 16% of the variance, CT+H 13%, CT 14%, and CT+H+D 5%.

After that, linear discriminant analysis effect size (LEfSe) was executed to establish the taxa that drive the differences between treatments (**Figure S2**). The ANCOM-BC differential analysis was exploited to pinpoint the differentially abundant taxa between the treatments. These two methods achieve the same results but from a different perspective. In roots samples, through the LefSe analysis, 8 microbial biomarkers were identified in CT+H, 17 in CT+H+D, 7 in CT+D, 12 in CT and 1 in C, while in soil samples, the biomarkers were 2 (heat), 17 (stress combination), 15 (drought), 7 (*Trichoderma*) and 12 (control).

Notably, the most discriminant taxa in CT+H+D belonged to the *Pseudomonadales* while CT was characterized by the *Clostridiales, Azospirillales and Clostridium* in *sensu strictu* and the C by the *firmicutes.* Moreover, in the soil, *Rhizobiales, Caulobacterales, Streptomicetales* were the major taxa in combined stress, while the most discriminant taxa in *Trichoderma* samples (CT) were *Enterobacteriales*.

### MultiOmics integration

**Figure 7:**
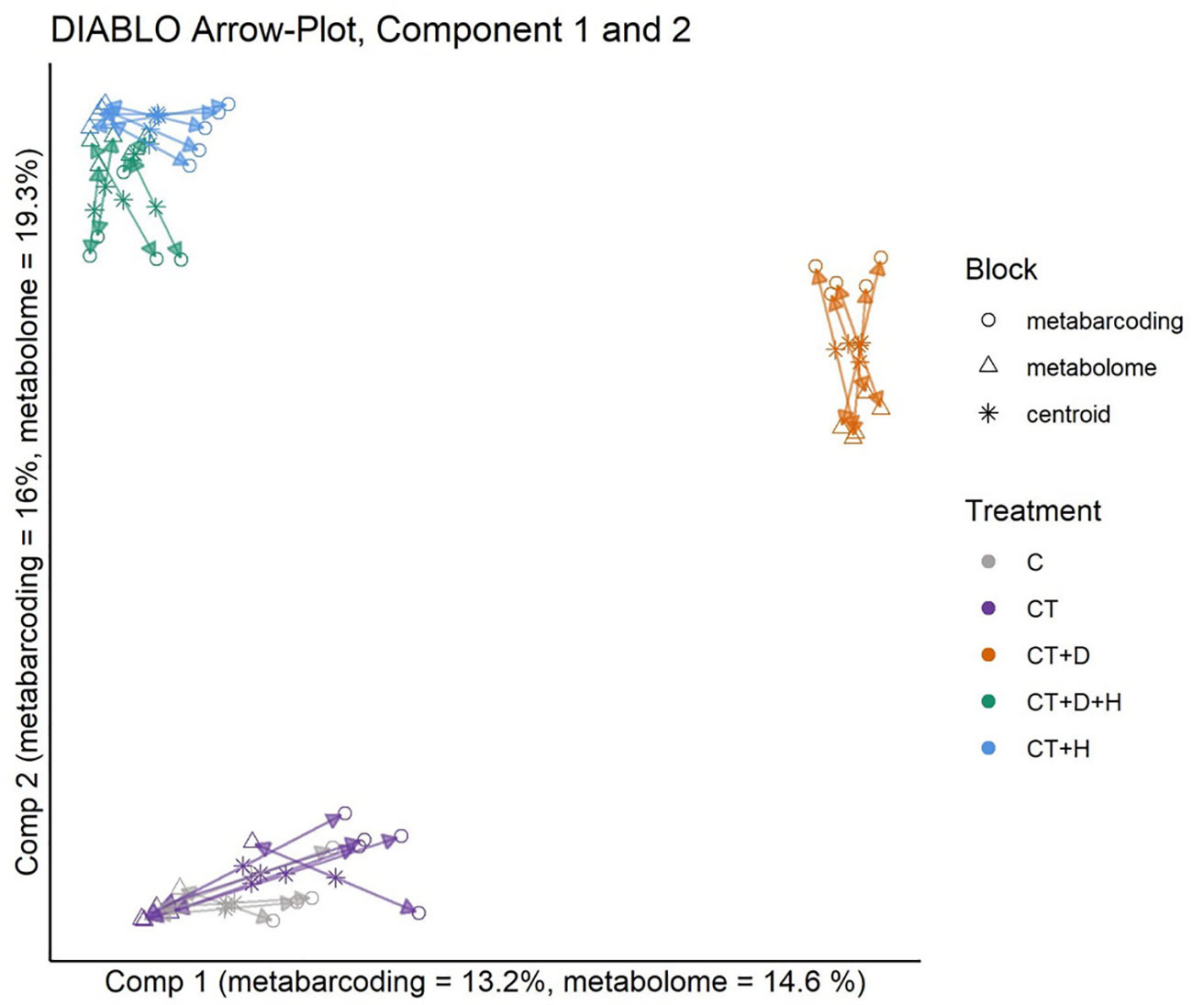
Arrow Plot from multiblock sPLS-DA (DIABLO): Samples of the data blocks metabarcoding and metabolomics are plotted into the space spanned by the first two components of the model. The length of the arrows indicates the distance of each sample from the centroids of both datasets. Short distances indicate high levels of agreement between metabolomics and metabarcoding blocks.

Using the DIABLO framework, the metabolomics and metabarcoding dataset from root samples were integrated (**Figure 7**). Model tuning indicated 4 components and a total of 102 for metabarcoding, and 130 metabolomics data would explain the most variance between treatments. The datasets are highly correlated for all 4 components. The metabarcoding and metabolomics were highly in agreement for all samples and treatments. On the second component, the CT+H, CT+H+D and CT+D were separated from the C and the CT. Looking at components 3 and 4 (**Supplementary Figure S3**), C and CT were mostly separated on component 3, while CT+H and CT+H+D were the most distant on component 4. Pearson correlation network analysis was performed as a follow-up analysis to DIABLO to identify positive correlations between features assigned to the same treatment by DIABLO. These pairs likely resolve the underlying interaction between the plant metabolome and the rhizosphere microbiota. Only bacterial genera and plant metabolites significantly assigned to a feature by DIABLO were included in network construction. Three clustering methods (Optimal, Louvain, and fast greedy clustering) reproduced four similar modules. Correlations between features of the control treatment and several heat or drought metabolites mainly constituted the largest. The latter was likely a false positive of the DIABLO analyses.

## Discussion

The use of *Trichoderma* spp. in horticulture is increasing due to its positive effects on crop plants and its high efficacy as a biofertilizer. The fungus application on seeds, leaves or in the soil reduces plant diseases, activates plant defense and tolerance to abiotic stress (i.e. drought, salinity, low temperatures, and pollutants) (Şesan et al., 2020). Although most of the studies on *Trichoderma* spp. were mainly focused on modifications induced on plant transcriptome and proteome, recent studies highlighted an improved production of secondary metabolites due to *Trichoderma* application (Fiorentino et al., 2018). Nonetheless, little information is available concerning the host mechanisms involved in connecting the *Trichoderma* root colonization to the metabolite dynamics, leading to increased plant development and/or activation of plant resistance to stress. It is thought that *Trichoderma* triggers a signaling cascade that causes a range of responses, including the accumulation of defense-related specialized metabolites also characterized by plant growth-promoting activity (Stewart and Hill, 2014; Zaidi et al., 2014).

In our experiments, plants treated with T were characterized by an increment of the fresh biomass even during D and H stress. Only the combination of D+H biomass was comparable to the control level. On the contrary, no differences were observed in DW parameter, whereas the DW/FW ratio was significantly reduced in all treatments except for C and CT+H+D. The reduction in this parameter sustained in the presence of T alone or during H and D stress suggests that the increase in FW observed was mainly due to an accumulation of water inside plant tissues. These results agree with several studies which suggest that *Trichoderma* increase drought tolerance in plants, enhancing a more efficient water use (Chepsergon et al., 2012). This hypothesis was further confirmed by plants treated with T to maintain a low leaf temperature even during D stress application. Plants exposed to D stress are generally characterized by a reduction in biomass production (reduced FW and DW), a decrease in chlorophyll content (reduction of SPAD units), and an increase in leaf temperature. These are typical symptoms of drought stress where plants, to cope with it, reduce stomatal conductance, decreasing the water losses and causing a reduction of the net photosynthesis and, as a consequence, the biomass production if the stress continues for a long time (Wang et al., 2010). Moreover, the increase in leaf temperature, mainly due to the reduced transpiration induced by the stomatal closure, could cause side effects on the photosynthetic machinery, inducing ROS accumulation (i.e. superoxide anion) and physical damages to the antenna complex, highlighted by Fv/Fm reduction, as also observed in our experiments. On the other side, the treatment with *Trichoderma* was characterized by a duplex effect. From one side, in control condition, the inoculated plants highlighted a higher biomass production and an increase of chlorophyll content, confirming its biostimulant/growth-promoting action, widely observed in the literature (Azarmi et al., 2011; Samia Ageeb Akladious, 2012). On the other side, during stress conditions (D and D+H), *Trichoderma* treatment further confirmed its protective role (Chepsergon et al., 2012; Estévez-Geffriaud et al., 2020; Scudeletti et al., 2021), supporting the plant during the stress phase and mitigating its effects (lower leaves temperature, higher biomass, and reduced damages to PSII).

Accordingly, in our experimental conditions, the inoculation with *Trichoderma* leads to increased production of phenylpropanoids, phytoalexins, glucosinolates biosynthesis, and modifications in the phytohormones profile (Iula et al., 2021). Similarly (Luo et al., 2010), demonstrated that the local application of trichokonin, a specialized metabolite produced by *Trichoderma* spp., on tobacco leaves acted as an elicitor stimulating the production of phenolic compounds, ROS and virus resistance through multiple defenses signaling pathways. Enhanced flavonoids, lignans, flavones, and flavanones production might help the plant to acquire more nutrients through root growth and change the plant microbiome (Voges et al., 2019): in the rhizosphere, these molecules act as chemo-attractants, inducers for rhizobia, cell receptors, and can inhibit the nitrification and modulate enzymes (Lucini et al., 2019). In addition, flavonoids have antimicrobial activity against many pathogenic microorganisms but are also a quorum sensing inducer for communications among microbes (Górniak et al., 2019).

Also, a strong accumulation of terpenes was found in CT+D, CT+H and CT, while an opposite trend was observed considering the CT+H+D, underlighting a different plant response under the combined stresses. Terpenoids are the major class of secondary metabolites formed from derivatives of glycolytic or acetyl CoA intermediates and are the component of several sterols, pigments and phytohormones (Jan et al., 2021). Moreover, besides playing a vital role in plants' defense mechanisms, they are one of the specialized metabolites that regulate the growth of specific root bacteria (Wang and Niu, 2019). Notably, terpenes selectively increase the proliferation of *Proteobacteria* strains while *Actinobacteria* are inhibited, underlighting the role of these compounds in selecting Arabidopsis root microbes. Furthermore, the exogenous application of the fungus in stressed plants also affected the phytoalexin content. These compounds might be produced *via* the phenylpropanoid pathway through the tryptophane and mevalonic pathways. These reactions involve the phytohormones auxins and abscisic acid (ABA), i.e the biosynthesis of numerous phytoalexins is downregulated by ABA (Bizuneh, 2021).

Overall, modulation of nitrogen-containing compounds, including glucosinolates and alkaloids, was observed. The accumulation of glucosinolates has been reported as a plant's osmotic adjustment process under drought conditions (Martínez-Ballesta et al., 2013). However, contradictory results were observed depending on the duration of the drought and the plant's development stage, with no effect on their concentration under high and mild drought (Martínez-Ballesta et al., 2013). Similarly, under high temperatures, increased glucosinolates levels were documented. Still, their variation depends on the genotype, the growth environments, the plant’s developmental stage and the interaction with other factors (i.e., light intensity) (Charron et al., 2005). Poveda et al., highlighted that the ability of *Trichoderma* to colonize the roots of *A.thaliana* is related to its capability to tolerate the presence of glucosinolates in the roots and demonstrated that despite the low content of glucosinolates, the application of *Trichoderma*, lead to greater tolerance to abiotic stresses (Poveda et al., 2019; Poveda, 2021).

In Arabidopsis, *Trichoderma* spp. were found to secrete auxin-related substances (i.e., indole-3-acetaldehyde, indole-3-carboxaldehyde, and indole-3-ethanol), small peptides, and volatile organic compounds, which improve root system architecture and solubilization/assimilation of macro and micronutrients (Abdullah et al., 2021). Under stress, auxins, cytokinins, ethylene, gibberellins and jasmonates were altered after *Trichoderma* treatment. Evidence for the role of Jasmonic acid and ethylene in the resistance induced by *Trichoderma* was provided (Martínez-Medina et al., 2014). During the *Trichoderma*–plant interaction, the fungi can enhance the content of endogenous defense and growth-related plant hormones (Sofo et al., 2011). Notably, the phytohormonal signaling is influenced by the beneficial soil microbes, which contribute to increased stress tolerance in plants. Therefore, increasing cytokinins in response to D and H+D were outlined. Cytokinins have both positive and negative effects on stress tolerance. In fact, several studies found decreased concentrations of cytokinins in response to extended stress, but conversely, some others reported that short-severe stress increased the cytokinin levels (Zwack and Rashotte, 2015). In *Arabidopsis*, jasmonic acid regulates stomatal movements. Its endogenous concentration promptly increases during drought stress and then returns to the early levels if the stress is prolonged (Wasternack and Hause, 2013).

Likewise, *Trichoderma* spp. was found to interact with the microbial community in the rhizosphere (Fiorentino et al., 2018). The plant holobiont concept assumes that *i)* the microbiome composition is strictly environmental-dependent, *ii)* that the plant may have selected some host-adapted microbes because they supply *ad hoc* advantages, and *iii)* that there is growing evidence that microorganisms can bring significant phenotyping modifications (Vandenkoornhuyse et al., 2015). Therefore, the plant can no longer be considered a standalone entity. Bacteria belonging to different genera i.e. *Pseudomonas*, *Bacillus*, *Rhizobium*, *Burkholderia*, *Azospirillum* and *Enterobacter* provide tolerance to the host plants under stress with the production of cytokinins, indole acetic acid, gibberellins and antioxidants (Grover et al., 2011) and providing additional genes to the host, which are involved in the adjustment to environmental conditions (Vandenkoornhuyse et al., 2015). Notably, some root endophytes can affect plant hormones by synthesizing auxins and their related compounds, and gibberellins, by using effectors (i.e mycorrhizal fungi) or by accumulating Jasmonic acid to avoid the salicylic acid triggered response.

According to our results, the soil microbiomes shifted in abundance and composition in response to environmental factors and *Trichoderma* treatments, also because the metabolites exuded by the plant shape the microbes communities resulting in different recruitment of microbial taxa (Korenblum et al., 2020). This modified rhizosphere microbial community might help alleviate the drought stress by producing exopolysaccharides or 1-aminocyclopropane-1-carboxylate (ACC) deaminase (Niu et al., 2018). Studies on fungal microbiome outlined the beneficial effects of fungi (arbuscular mycorrhiza, AM) in improving drought resistance (Xu et al., 2018). The bacterial community in the root system respond to early drought delaying the development of the root and the rhizosphere microbiome and leading to an enrichment of gram-positive bacteria, including Firmicutes (Maclean et al., 2017).

## Conclusion

An effective and environmentally friendly way to increase crop production and maximize resource use efficiency, minimizing the environmental effects on the ecosystem, might be the exploitation of biostimulants. *Trichoderma* spp. are widely used due to their growth-stimulating effect and control towards rhizospheric pathogens. In this work, the application of *Trichoderma* spp. displayed a reprogramming of secondary metabolism and phytohormones profile along with changes in roots and rhizosphere microbial population. The secreted hydrolytic enzymes and secondary metabolites by *Trichoderma* spp. improve biotic and abiotic stress tolerance and plant defenses inducing root development, seed germination, increasing chlorophyll content and photosynthetic efficiency. Through the correlation-based analysis of untargeted metabolomics and metabarcoding, an integrated knowledge about the metabolic networks that regulate plant stress responses and how plant secondary metabolites changed plant-root microbiomes was achieved. Through such an multi-omics approach, it also might possible in future studies to identify root microbiomes influence on important plant traits such as growth abiotic stress tolerance, resistance to infectious diseases, and the synthesis of phytohormones.

## Data availability statement

The data presented in the study are deposited in the Sequence Read Archive (SRA) as part of BioProject PRJNA900448, accession numbers SAMN31692959-SAMN31692963, SAMN31692979-SAMN31692983 and SAMN33924422-SAMN33924461.

## Author contributions

BS drafted the first manuscript draft and conducted laboratory work. BS, AF, LL and SK conceptualized the study and supported writing the manuscript. AF supported the plant pot experiment. SL conducted multi-omics synthesis analyses. SW conducted and supported bioinformatic analyses associated with metabarcoding. All authors contributed to the article and approved the submitted version.
