# Supplementary figures and images for: Trichoderma spp.-mediated mitigation of heat, drought, and their combination on the Arabidopsis thaliana holobiont: a metabolomics and metabarcoding approach
Source: Front Plant Sci. 2023 Aug 21;14:1190304. doi: 10.3389/fpls.2023.1190304 (PMC10484583; doi:10.3389/fpls.2023.1190304)

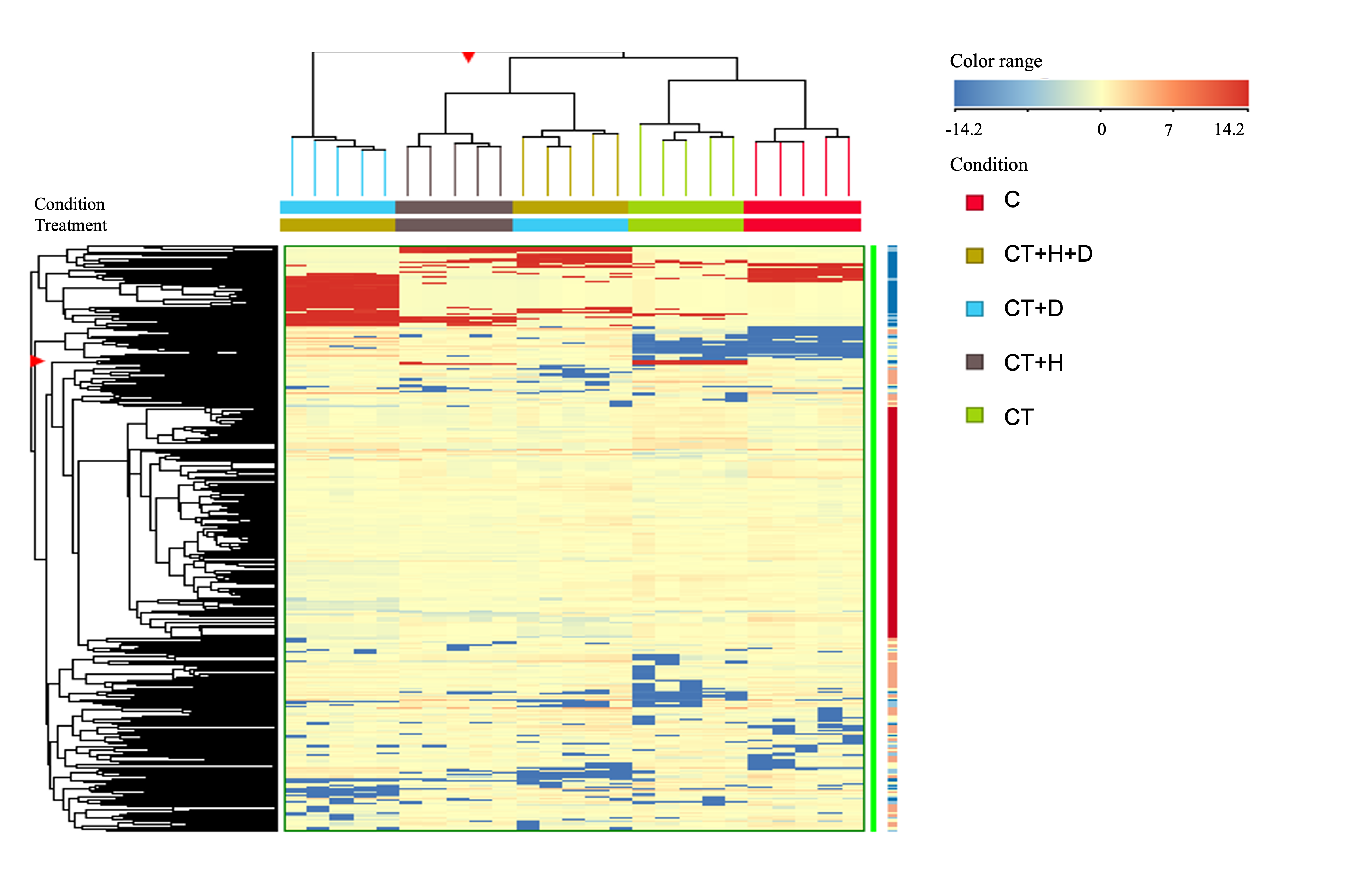

Supplement: Supplementary file 1 [file Image_1.png]

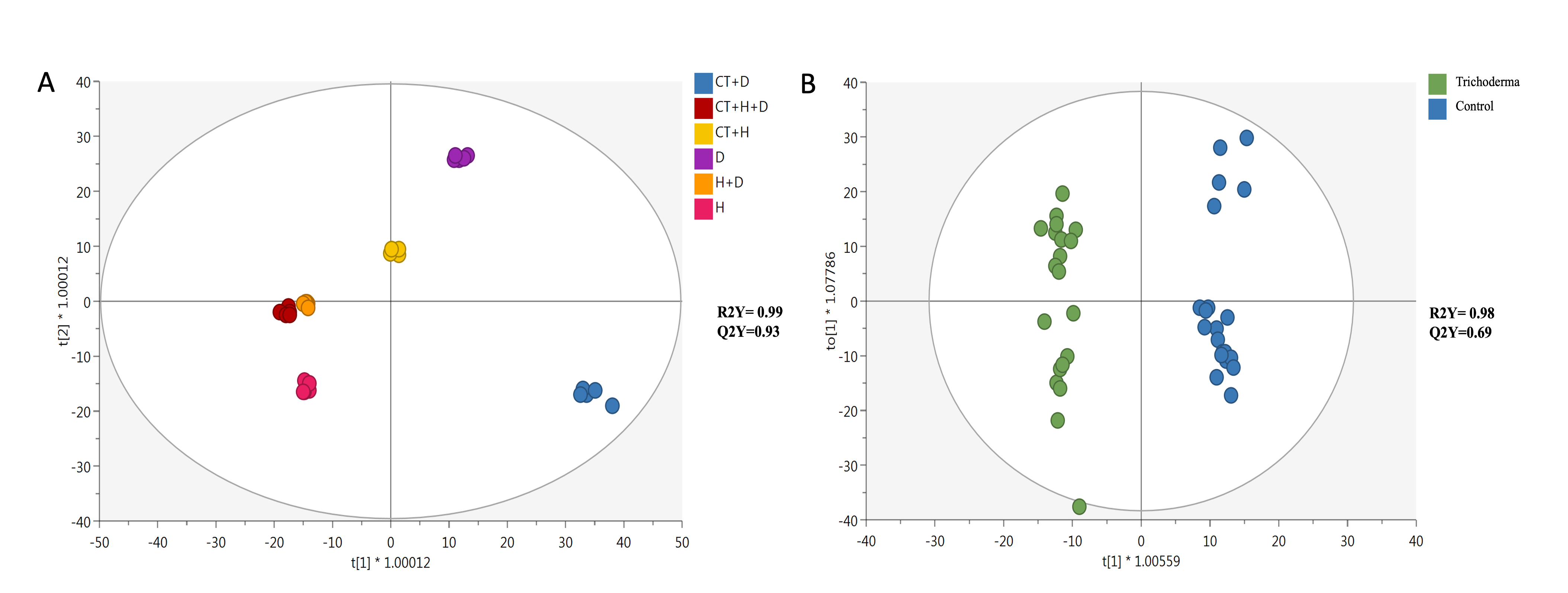

Supplement: Supplementary file 2 [file Image_2.png]

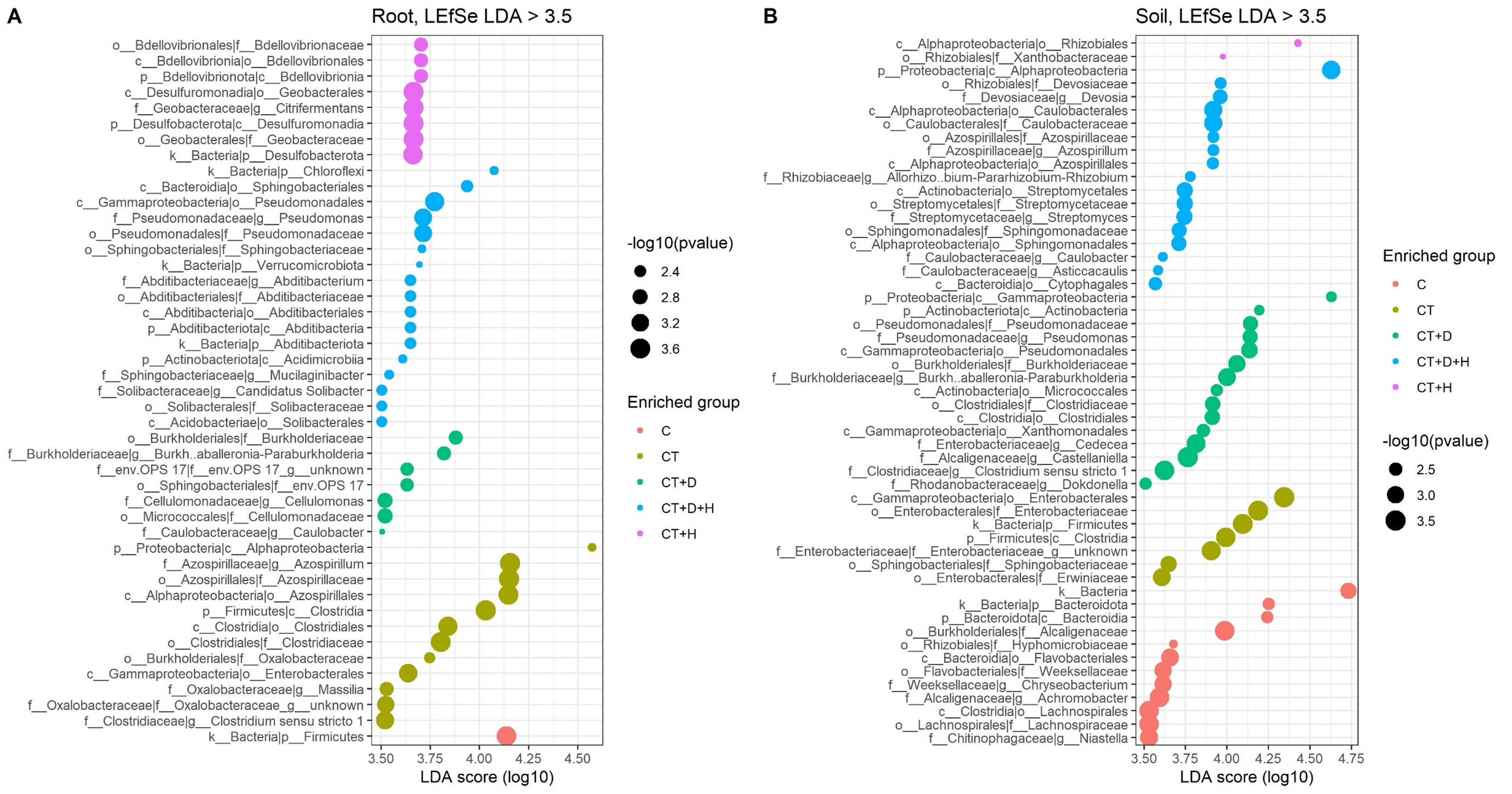

Supplement: Supplementary file 3 [file Image_3.png]

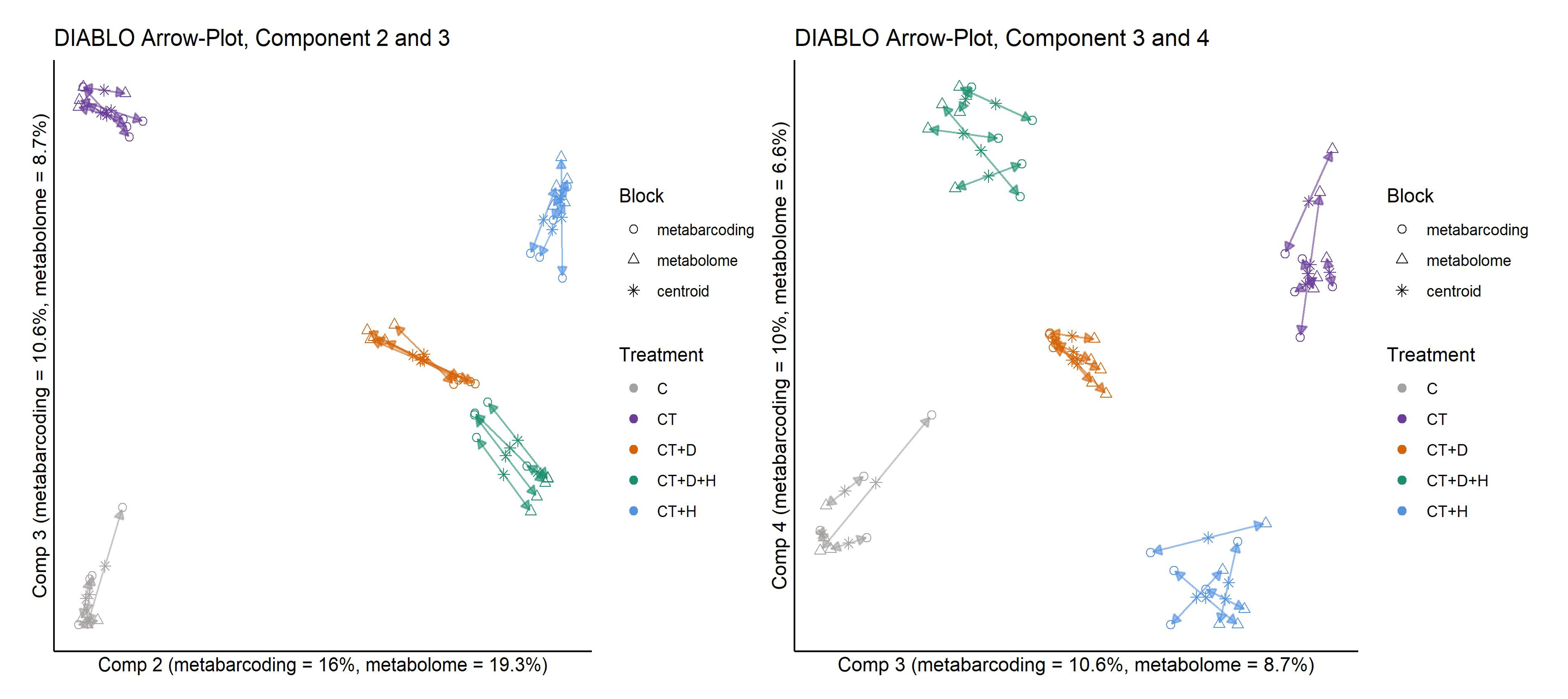

Supplement: Supplementary file 4 [file Image_4.png]
