# Supplementary material for: Trichoderma spp.-mediated mitigation of heat, drought, and their combination on the Arabidopsis thaliana holobiont: a metabolomics and metabarcoding approach
Source: Front Plant Sci. 2023 Aug 21;14:1190304. doi: 10.3389/fpls.2023.1190304 (PMC10484583; doi:10.3389/fpls.2023.1190304)
Supplement: Supplementary file 5 [file Table_1.docx]

Supplementary Material

*Trichoderma spp.*-mediated mitigation of heat, stress, and their combination on the *Arabidopsis thaliana* holobiont: a metabolomics and metabarcoding approach

Biancamaria Senizza^1^, Fabrizio Araniti^2^, Simon Lewin^3^, Sonja Wende^3^, Steffen Kolb^3,4*,^ Luigi Lucini^1^

*** Correspondence:** Steffen Kolb: Kolb@zalf.de

1. **Supplementary Tables**

**Supplementary Table S1.** The two-way ANOVA based on the morpho-physiological parameters considering both *Trichoderma spp.* treated samples and their corresponding controls (not-treated).

**Supplementary Table S2.** Whole dataset produced from untargeted metabolomics carried out in *Arabidopsis* *thaliana* roots treated with and without *Trichoderma spp*. and exposed to heat, drought and the combined stress. Compounds are presented with individual intensities and with composite mass spectra (monoisotopic accurate mass/abundance combinations).

**Supplementary Table S3.** Discriminant metabolites (VIP markers) identified by the OPLS-DA analysis in *Arabidopsis thaliana* roots exposed to heat, drought and combined stress.

**Supplementary Table S4**. Differential metabolites derived from ANOVA and fold-change (FC) analysis (p-value < 0.05, Benjamini multiple testing correction; fold-change threshold FC ≥ 2) in roots metabolomic profiles after stress exposure. These compounds were uploaded into the Omic Viewer Pathway Tool of PlantCyc (Plant Metabolic Network, <http://www.plantcyc.org/>).

**Supplementary Table S5.** Bray-Curtis dissimilarity indices were calculated on rarefied relative abundances and used to perform principal coordinate analysis (PCoA) and permutational analysis of variance (PERMANOVA) to investigate treatments' effect on the bacterial community structure.

## Supplementary Figures

**Supplementary Figure S1.** Unsupervised hierarchical cluster analysis (Euclidean distance; linkage rule: Ward) carried out from root chemical profiles treated with *Trichoderma spp*. and exposed to heat, drought and the combined stress. Metabolites were obtained by UHPLC-ESI/QTOF-MS untargeted analysis, and their intensities used to build up the fold-change heatmap here provided.

**Supplementary Figure S2:** The score plots of orthogonal projection to latent structures discriminant analysis (OPLS-DA) supervised modeling carried out on untargeted metabolomics profiles of *Arabidopsis thaliana* roots exposed to heat, drought, and combined stresses and treated with *Trichoderma spp.*

**Supplementary Figure S3**: Plotted effect Size of Biomarkers identified by LefSE analysis for A) root and B) soil with an LDA score over 3. Color indicates the treatment they discriminant for size of dot represent p-value. The marker taxonomic lineage is displayed on 3 levels.

**Supplementary Figure S4**: multiblock sPLS-DA (DIABLO). Samples from data blocks metabarcoding and metabolomics are plotted into space spanned by the second and third components. Length of the arrows indicate the distance of each sample from the centroids of both datasets. Short distances show a high level of agreement between blocks. For all samples and treatments, the agreement between metabarcoding and metabolomics is high.
